# The Impact of Zinc and Zinc Homeostasis on the Intestinal Mucosal Barrier and Intestinal Diseases

**DOI:** 10.3390/biom12070900

**Published:** 2022-06-27

**Authors:** Yan Wan, Bingkun Zhang

**Affiliations:** State Key Laboratory of Animal Nutrition, College of Animal Science and Technology, China Agricultural University, Haidian District, Beijing 100193, China; wy1208@cau.edu.cn

**Keywords:** zinc, zinc homeostasis, intestinal mucosal barrier, inflammatory bowel disease, irritable bowel syndrome, colorectal cancer

## Abstract

Zinc is an essential trace element for living organisms, and zinc homeostasis is essential for the maintenance of the normal physiological functions of cells and organisms. The intestine is the main location for zinc absorption and excretion, while zinc and zinc homeostasis is also of great significance to the structure and function of the intestinal mucosal barrier. Zinc excess or deficiency and zinc homeostatic imbalance are all associated with many intestinal diseases, such as IBD (inflammatory bowel disease), IBS (irritable bowel syndrome), and CRC (colorectal cancer). In this review, we describe the role of zinc and zinc homeostasis in the intestinal mucosal barrier and the relevance of zinc homeostasis to gastrointestinal diseases.

## 1. Introduction

Zinc is the second most abundant trace element in the body and is a catalytic cofactor for many enzymes or a structural cofactor for proteins. Indeed, more than 300 enzymes and 1000 transcription factors rely on zinc to perform their biological functions [1,2]. Zinc is essential for various physiological functions, such as growth, development, antioxidant activity, and immunity [3,4]. Additionally, Zn^2+^ is involved in the regulation of cellular signal pathways as a signal molecule [5,6].

Plasma or serum zinc levels in healthy individuals vary from 12 to 16 µM, which corresponds to less than 1% of the whole-body zinc [7]. Both insufficient and excessive zinc can disrupt zinc homeostasis; zinc deficiency dysregulates cellular function, and excess zinc is also toxic to cells. Thus, the levels of zinc are necessarily tightly fine-tuned to maintain zinc homeostasis in organisms.

On the one hand, zinc homeostasis refers to the stabilization of zinc in body fluids, tissues, and organs, which mainly depends on the uptake and excretion of zinc in the body. On the other hand, it refers to the stability of intracellular zinc, which depends on various zinc homeostasis-related proteins, such as zinc transporters and zinc binding proteins. Zinc transporters consist of two families: the ZIP (Zrt- and Irt-like proteins, also known as SLC39A) family and the ZnT (Zn transporter, also known as SLC30A) family. At present, the ZIP family contains 14 members in mammals, and its members transport zinc from the extracellular space and intracellular organelles to the cytoplasm. The ZnT family contains 10 members, and they transport zinc from the cytoplasm to extracellular space or organelles [8,9,10]. The coordination of the two families’ members maintains the homeostasis of intracellular zinc. Metallothionein (MT) is a cysteine-rich metal-binding protein in cells and is the most important zinc-binding protein [11]. It is both a zinc receptor and a zinc donor, which can regulate the absorption, distribution, storage, and release of zinc in the cells.

The intestinal mucosal barrier is composed of an extracellular mucus layer and intestinal epithelial cells (IECs). Functionally, the intestinal mucosal barrier can be divided into a mechanical barrier, chemical barrier, biological barrier, and immune barrier. It not only electively absorbs nutrients from the chyme and transfers them from the intestinal lumen to the metabolic cycle, but it also plays an indispensable role in defending against microorganisms and exogenous antigens. Studies have demonstrated that a variety of intestinal diseases are associated with intestinal mucosal barrier damage, including inflammatory bowel disease (IBD), irritable bowel syndrome (IBS), and colorectal cancer (CRC) [12,13,14].

Many lines of evidence indicate that zinc homeostasis is essential for the maintenance of intestinal mucosal barrier functions, and they have efficacy in some intestinal disorders. Zinc deficiency causes mucosal barrier dysfunction [15], while zinc supplementation improves these symptoms [16]. In this review, we focus on the roles of zinc and zinc homeostasis in the intestinal mucosal barrier, as well as their functions and mechanisms in the occurrence and development of intestinal diseases.

## 2. Zinc Homeostasis in the Intestinal Mucosal Barrier

The adult human body contains approximately 2.6 g of zinc, and the fraction in the intestine is about 1.2% [7]. The small intestine is the main place for the absorption and excretion of zinc [7,17]; thus, an injury to the intestinal mucosa can affect the absorption and excretion of zinc, thereby affecting the zinc homeostasis in cells and organisms. Correspondingly, the imbalance of intestinal zinc homeostasis can also negatively affect the structure and function of intestinal mucosal barrier.

### 2.1. Zinc Absorption and Excretion

Exogenous zinc is taken up at the intestinal brush border membrane, where it is transported from the lumen to enterocytes; subsequently, the cation is excreted at the basolateral side of the enterocytes to release the Zn^2+^ into the portal blood, which distributes Zn^2+^ in the body [17,18]. After circulating in the body, zinc is excreted into the small intestinal lumen along with pancreatic secretions and bile; most of the zinc is reabsorbed by the small intestine, and the remaining part is excreted in feces [19]. In cells, zinc exists in three forms: zinc bonded to proteins, zinc stored in vesicles, and cytoplasmic free zinc [17]. The absorption of zinc in the small intestine occurs in two ways: passive transport through ion-channels and active transport dependent on zinc transporters.

**Figure 1:**
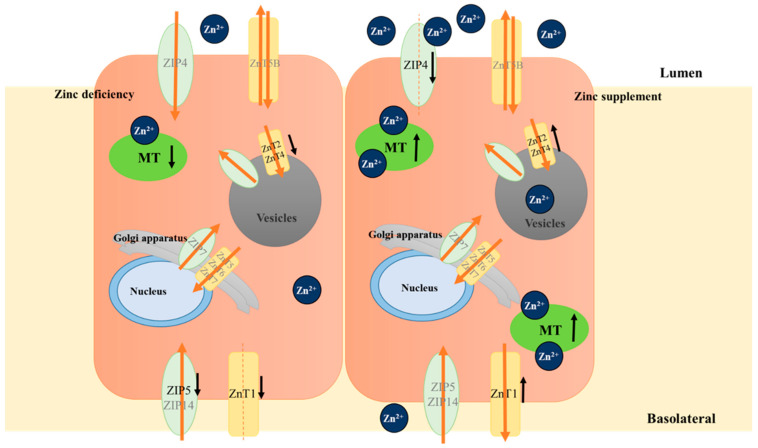
Zinc transporters (ZnT and ZIP) and zinc-binding proteins (MT) are involved in the regulation of intestinal zinc absorption, a potential regulatory mechanism of zinc absorption into enterocytes during zinc excess and zinc supplement. The red arrows indicate the direction of zinc flow, and the black arrows indicate changes in zinc transporters’ RNA and/or protein upon changes in the zinc state.

Zinc transporters consist of two families: the ZIP family and the ZnT family. Corresponding to the wide distribution of zinc, the distribution of zinc transports in tissues and organs or the localization in cells show specificity and diversity. Intestinal zinc absorption is mainly mediated by ZnT1, ZnT2, ZnT5, ZnT6, ZnT7, and ZnT5B (a splicing variant of ZnT5) of the ZnT family and ZIP1, ZIP4, ZIP5, ZIP10, and ZIP14 of the ZIP family [20,21]. In addition, MT is also involved in the regulation of enterocyte zinc homeostasis through binding and releasing cytoplasmic free Zn^2+^ in cells [11] (as shown in Figure 1).

ZIP4 is localized on the apical membrane of enterocytes and is the main transporter of Zn^2+^ from the lumen to enterocytes. Studies have found that the ZIP4 gene mutations cause zinc absorption disorder and acrodermatitis enteropathica [22]. ZIP4 translocates to the apical membrane during zinc deficiency, thereby increasing Zn^2+^ uptake by enterocytes [23,24,25]. Under excess zinc conditions, the ZIP4 on the cell surface is rapidly endocytosed, ubiquitinated, and degraded [24,26]. When zinc is sufficient, ZIP5 is localized on the basolateral membrane of enterocytes and participates in cellular zinc excretion. When zinc is deficient, the ZIP5 on the basolateral membrane of enterocytes is endocytosed and degraded to reduce the zinc from the blood circulation to enterocytes [24,25]. ZIP14 is also localized on the basolateral membrane of enterocytes and participates in the excretion of cellular zinc [27], but its protein expression is generally insensitive to the zinc status of the cells.

Different from the ZIP family, there are only ZnT1 and ZnT5B localized on the cell membrane of the ZnT family, and most of the others are located in the organelle and nucleus. ZnT1 is located on the basolateral membrane of enterocytes, and it is responsible for the transport of Zn^2+^ from the enterocyte to the extracellular space [28]. The expression of ZnT1 in the intestine at the mRNA and protein levels increases when zinc is sufficient [29] and decreases during zinc deficiency [29,30]. ZnT5B is a splice variant of ZnT5, which is located on the apical membrane of enterocytes. It is capable of the bidirectional transport of zinc [31]. In the IECs, ZnT2 and ZnT4 mainly accumulate in vesicles [28], their gene expression increases under the condition of sufficient zinc, which mediates zinc transport into vesicles and isolates it from the cytoplasm, while under zinc depletion, their gene expression decreases [29]. ZnT6 is found in the ER and Golgi apparatus, but it is incapable of transporting zinc alone; instead, it is capable of transporting zinc by forming heterodimers with ZnT5 and its orthologs [32]. ZnT7 accumulates zinc in the Golgi of cells; thus, it is an important transporter for zinc transport in the Golgi [33]. Studies have shown that zinc depletion in HeLa epithelial cells results in increasing mRNA expression of ZnT5 and ZnT7 [34]; zinc supplementation reduces the mRNA levels of ZnT6 and induces ZnT7 in THP-1 mononuclear cells [35], and in zinc-deficient Raji (B cell line), the expression of ZnT5, 6, and 7 is improved [36]. Nevertheless, in intestinal cells, no study has shown whether zinc homeostasis affects the expression of ZnT5, 6, 7. In addition to zinc transporters, the expression of MT is also closely related to zinc homeostasis. Data have indicated that a high zinc supply can induce an increase in cellular MT in vivo and in vitro [37]. MT is able to bind and release Zn^2+^ which is absorbed into the cells; consequently, changes in MT expression may affect zinc transport kinetics and luminal zinc absorption [17].

In conclusion, specific zinc transporters and zinc-binding proteins work together to maintain the dynamic balance of zinc metabolism in IECs and maintain body zinc homeostasis by regulating zinc absorption and excretion.

### 2.2. Interactions between the Zinc Homeostasis Imbalance and Intestinal Mucosal Barrier Injury

A zinc deficiency can injure the intestinal mucosal barrier. The study of Zhong et al. [15] showed that treating Caco2 cells with TPEN for zinc deprivation resulted in decreased expression of tight junction proteins, increased intestinal epithelial permeability, and impaired intestinal mucosal barrier function. An in vitro study by Miyoshi et al. [38] also found that the tight junction permeability in Caco2 cells increased in the presence of zinc deficiency, and cell viability was significantly reduced. Furthermore, the repair and regeneration of the intestinal mucosa requires the participation of zinc; so, exogenous supplementation can maintain the integrity of the intestinal mucosal barrier morphology and function [39]. These results suggest that zinc plays an important role in maintaining the intestinal barrier function.

Individuals with a compromised intestinal mucosal barrier develop an imbalance in zinc homeostasis. A recent meta-analysis study demonstrated a significant relationship between serum zinc levels and Crohn’s disease (CD), which is characterized by injured intestinal mucosa [40]. The imbalance of zinc homeostasis is due to the absorption of zinc in the small intestine being reduced and the endogenous zinc excretion being increased when the intestinal mucosal barrier is damaged, and the intestinal permeability is decreased. Griffin’s study [41] found that plasma zinc levels were reduced in CD patients, and the absorption of zinc in the small intestine was also reduced, while endogenous zinc excretion did not change significantly. However, Main et al. [42] found CD patients’ zinc excretion decreased; this difference may be related to the course of CD. In addition to peripheral blood zinc levels, a study also found that ZIP8 mutation in CD patients was associated with changes in intestinal flora [43], suggesting that zinc homeostasis in enterocytes is also important for maintaining the intestinal mucosal barrier.

## 3. Mechanism of Zinc Homeostasis Affecting the Intestinal Mucosal Barrier

### 3.1. Intestinal Mucosal Physical and Chemical Barriers

The physical barrier is composed of intestinal epithelial cells, intercellular junctions and the submucosal lamina propria, which can prevent the macromolecular substances and pathogens in the intestinal lumen crossing the intestinal wall. The intestinal epithelium is composed of crypt and villus, which are made up of several different types of cells, including intestinal epithelial stem cells, IECs, and enteroendocrine cells. The chemical barrier is formed by mucin, which is secreted by intestinal goblet cells and various antimicrobial proteins, which are secreted by IECs, which can prevent macromolecular substances from directly contacting the epithelial cell layer and defend against foreign antigen. Zinc and zinc homeostasis is crucial in the maintenance of the physical and chemical barriers. Studies have suggested that zinc supplementation increases the villus height and reduces the crypt depth in the mammalian and avian small intestine [44,45,46,47,48] and increases the number of intestinal goblet cells [49] and the expression of MUC2 [46,47,48].

Zinc transporters are involved in the continuous self-renewal of the intestinal epithelial cells and the maintenance of the overall structure of the crypt–villus axis. Ohashi et al. [50] illustrated that ZIP7 is essential for intestinal epithelial homeostasis by the regulation of the endoplasmic reticulum function in proliferative progenitor cells and the maintenance of stem cells in the crypt; Zip7 deficiency in the intestinal epithelium leads to the loss of Olfm4^+^ intestinal stem cells and the degeneration of post-mitotic Paneth cells. The findings provided important evidence for the role of ZIP7 in maintaining intestinal epithelial homeostasis. Furthermore, Geiser et al. [22] showed all Zip4 intestine knockout mice died within two to three weeks. In ZIP4 knockout mice, the expression of Sox9, a marker for Paneth cells decreased, and the lysozyme activity also decreased in the small intestine, accompanied by the disorganization of the crypt and villus morphology and significantly diminished cell division, and the epithelial cells had lost much of their columnar morphology.

**Figure 2:**
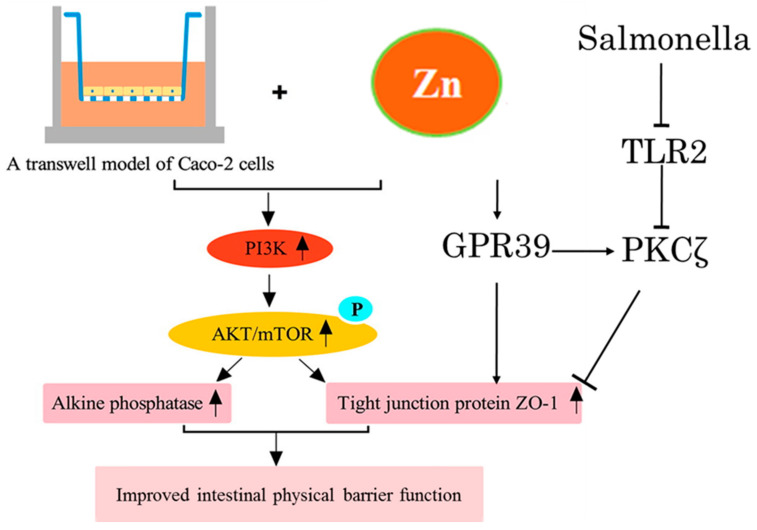
Cellular pathways in which zinc upregulates the function of the mucosal physical barrier. Zinc improves the expression of the tight junction protein by activating PI3K/AKT/mTOR. Zinc directly regulates the epithelial barrier via the G protein-coupled receptor (GPR39). Then, through the GPR39 molecule, it can activate PKCζ to alleviate the intestinal barrier dysfunction induced by *S. typhimurium*.

In addition to the regeneration and composition of IECs, zinc and zinc homeostasis also affects the tight junctions (TJ) between epithelial cells. Studies have shown that zinc deficiency results in the destruction of intercellular connections and increases intestinal permeability [38,51], while zinc supplementation can repair the injury of TJ. As the cellular pathways underlying this effect (Figure 2), the diosmectite–zinc oxide composite enhanced intestinal barrier restoration by modulating the TGF-β1, ERK1/2, and Akt pathways in acetic acid challenged piglets and weaned pigs [52,53]. Additionally, zinc was shown to have the known insulin-mimetic effects in activating the PI3K/protein kinase B (AKT) signaling cascade via the regulation of gene expression [54]. The activation of PI3K/AKT/mTOR signaling by zinc sulfate is involved in improving the intestinal barrier function by enhancing the cell differentiation and expression of TJ protein ZO-1 in Caco-2 cells [55]. In addition, extracellular Zn^2+^, via the G protein-coupled receptor (GPR39), directly regulates the epithelial barrier of the colon [54,56,57]. Shao et al. [58] found that zinc via the GPR39 molecule can activate PKCζ to alleviate the intestinal barrier dysfunction induced by *S. typhimurium*. Zinc transporters also contribute to maintain TJ. Gregory et al. [27] indicated that the intestinal permeability of ZIP14 knockout (ZIP14KO) mice was assessed to be higher than wildtype mice, and ZIP14KO mice showed a significant reduction in the amount of threonine-phosphorylated occludin and the expression of claudin1 and claudin2.

Zinc deficiency and supplementation affect the number of intestinal goblet cells and the number and composition of mucins [48,49,59]. An in vitro study showed that zinc deficiency affected goblet cell synthesis and the secretion of mucin, along with impairing O-glycan elongation, increasing the production of short sugar chains, and altering the core structure of O-glycans; this altered O-glycan pattern can affect the stability of the intestinal mucus layer [49]. Paneth cells are important sources of lysozyme and defenses in the intestine. Zinc is an essential trace metal for Paneth cells. Zinc coordination affords protection of human defensins against proteolytic degradation by the intestinal protease trypsin and other proteases [60], and ZnO nanoparticles has been shown to maintain the stability of lysozyme [61]. Similarly, the loss of function of some intestinal zinc transporters such as ZIP4 and ZIP7 can lead to a rapid loss of zinc in Paneth cells, which will stop the morphology and cell renewal of Paneth cells [22,50]. ZnT2 is the zinc transporter expressed on the secretory granules in Paneth cells; it can transport Zinc into Paneth cell granules to maintain granulin stability and secretory function [62]. Podany et al. [63] demonstrated in ZnT2Ko mice that the secretory granules were hypodense with less active lysozyme, and there was evidence of autophagosome accumulation and granule degradation in the Paneth cells from ZnT2ko mice.

To summarize, zinc and zinc transports maintain the physical barrier of intestinal mucosa by participating in the renewal of IECs, and the maintenance of the junction between intestinal epithelial cells. Moreover, zinc can also maintain the stability of the mucous layer and the production of antibacterial proteins, which contribute to the stability of the intestinal mucosal chemical barrier.

### 3.2. Intestinal Mucosal Immune and Biological Barriers

The intestinal mucosal immune barrier mainly consists of gut-associated lymphatic tissue (GALT), its secreted immunoglobulin A (sIgA), antimicrobial peptides, cytokines, and other immune active substances. It can identify abnormal antigens and induce an immune response to protect the intestine from being invaded and prevent excessive immune responses.

A study on poultry found that Zn–Gly chelate supplementation could balance the Th1 and Th2 response in the GALT through the activation of a cascade of cytokines released by stimulated T and B lymphocytes [64]. sIgA is secreted by plasma cells and serves as the first line of the immune barrier, which limits the access of antigens and bacteria in the intestinal tract to the circulation system [65]. Several studies have implied that zinc supplementation can upregulate the expression of sIgA [46,64,66]. Intestinal macrophages are the most abundant immune cell type in the lamina propria, which can clear both pathogens and apoptotic cells. MT can enhance the ability of autophagy and intracellular bacterial clearance through mediating the zinc accumulation of macrophages [67], while the synthesis and degradation of MT in macrophages are regulated by the concentration of cytoplasmic free zinc [68]. Hence, the zinc homeostasis maintained by MT plays a role in regulating the immune function of intestinal macrophages. Defensins are an important class of antimicrobial peptides with antimicrobial activity and are found widely in animals, plants, and fungi. They are an important part of host innate immunity. As previously mentioned, zinc homeostasis affects the morphology and function of Paneth cells [22,50,62,63], which are the main source of intestinal defensins. Some studies have indicated that exogenous addition of zinc can increase the gene expression of intestinal β-defensins in pigs and ducks [46,48]. A study has found the same in humans, the decrease in Paneth cell granules and reduced human defensin 5 (HD5) immunoreactivity are significantly associated with a decrease in the plasma zinc levels [69].

The intestinal mucosal biological barrier consists of the intestinal microbiota that restrict mucosal colonization by pathogens and resists penetration by pathogens.

Zinc deficiency caused by insufficient dietary zinc leads to poor zinc physiological status and induces a decrease in gut microbial diversity and an outgrowth of bacteria particularly suited to low zinc conditions, which eventually lead to an intestinal microecological disorder [70]. Experimental evidence has also shown that high dietary zinc supplementation alters the composition of the gut microbiota [71,72]. Zinc homeostasis affects intestinal microbial balance in two ways. First, as the above results show, zinc homeostasis has an impact on the function of immune cells and immune active substances, which play a crucial role in maintaining the balance of intestinal microorganisms; for example, macrophages have the ability to clear bacteria. Second, the growth and reproduction of gut microbes requires the participation of zinc. A study in mice colonized with *C. difficile* indicated that excess dietary zinc severely exacerbated C. *difficile*-associated disease by increasing its toxicity and altering the host immune response [71]. Based on this, the competition for zinc between the gut microbes and the host can be used to inhibit the infection of some pathogenic bacteria. The host can express some zinc-sequestering proteins, such as calprotectin, to the infection site to reduce the zinc concentration in a single location to limit zinc absorption by bacteria, thereby limiting the growth and virulence of pathogenic bacteria and inhibiting the proliferation of pathogenic bacteria against bacterial infection [73,74].

In conclusion, zinc can help maintain a functional mucosal immunological barrier by regulating the function of the intestinal immune cells and the expression of immune active substances. Simultaneously, zinc also can maintain the composition of gut microbes through immune mechanisms. In addition, zinc homeostasis of both host and microbes can affect the colonization and proliferation of gut microbes, as zinc is an essential component of microbial proteins.

## 4. Research Advances in the Relationship between Zinc Homeostasis and Gut Diseases

### 4.1. Inflammatory Bowel Disease

IBD is a nonspecific chronic inflammatory disease of the intestine, mainly including ulcerative colitis (UC) and CD. UC is usually confined to colonic tissue, with the occurrence of crypt destruction, erosions, and ulcers with diffuse mucosal inflammation and neutrophils [75]. Compared with UC, CD is an intestinal inflammation that can affect any part of the gastrointestinal tract, but the small intestine is the most common area for CD occurrence, especially the terminal ileum and colon [76]. At present, the pathogenesis of CD and UC are still inconclusive, and it is generally believed that they are related to genetic factors, environmental factors, infectious factors, and immune factors [77,78]. Studies have shown that CD and UC patients have a lower zinc intake and serum zinc levels [41,79,80], and both CD and UC patients with serum zinc deficiency are more likely to have adverse disease-specific outcomes, as these outcomes improve with normalization of zinc levels [80]. However, it is noteworthy that a meta-analysis study found that low serum zinc levels were significantly associated with CD but not with UC [40]. Similarly, a study by Ananthakrishnan et al. [81] also found that dietary zinc supplements were negatively correlated with CD risk but not with UC. Therefore, the relationship between CD or UC progression and zinc level concentration remains controversial, and more research needs to be conducted in the future.

Recently, there has been increasing attention to the mechanism of zinc’s effect on the occurrence and development of IBD. Yasuki [82] found that zinc deficiency activated Th17 cells to aggravate colitis, and the activation resulted from the induction of IL-23 expression in macrophages. In addition, zinc deficiency can also lead to lymphoid tissue hypoplasia, decreased NK cell activity, and increased apoptosis of B and T cells, thus affecting the production of antibodies and cell-mediated immunity [83], which can reduce the ability of antivirus and antibacterial activity and the body’s immunity and increase the risk of inflammation. Zinc deficiency can also lead to impaired intestinal barrier integrity and leaky gut, enabling pathogenic bacteria and other antigenic substances in the intestine to directly contact IECs, which can stimulate continuous submucosal inflammation [38,51]. Zinc supplementation can effectively reduce the penetration of bacteria through the intestinal mucosal barrier to avoid direct contact with IECs and resist the infection by enteric pathogens, thus reducing intestinal inflammation and improving diarrhea symptoms [53,57,84]. In short, the imbalance of zinc homeostasis increases the risk of impaired intestinal barriers, causes foreign antigens to be more likely to contact IECs directly, increases the risk of infection, and induces intestinal inflammation. It can also lead to the dysbiosis of intestinal flora and the imbalance of organism immunity, which ultimately leads to intestinal inflammation and promotes the onset and progression of CD or UC.

### 4.2. Irritable Bowel Syndrome

IBS is a common gastrointestinal disorder, which is characterized by chronic or recurrent abdominal pain, in conjunction with relief or aggravation with defecation or altered bowel habits [85]. The etiology and pathogenesis of IBS are not completely clear; it is generally recognized that IBS is a disease closely related to the central nervous system or psychosocial factors, food allergies, gastrointestinal changes, and genetic susceptibility [86]. The central nervous system or psychosocial factors are mainly related to visceral hypersensitivity, abnormal brain–gut axis interaction, and psychosocial dysfunction. Gastrointestinal changes mainly include an imbalance in intestinal flora, a motility disorder in the gastrointestinal tract, low-grade intestinal inflammation, and abnormal immune activation of intestinal mucosa.

A few studies are currently available on the role of zinc in IBS. Studies have found that the dietary zinc intake in IBS patients is significantly lower than those of healthy individuals, but there is no difference in the zinc levels in the plasma or serum [87,88]. A study of diarrhea-predominant irritable bowel syndrome (IBS-D) patients suggested that the serum zinc levels and odds of IBS-D were inversely associated [89]. Abnormal copper–zinc ratios have been found in many mental health disorders that overlap with IBS [90,91]. A North American study also found that the serum copper–zinc ratio was increased in IBS patients, but there was no significant difference in serum copper or zinc levels [92]; the possible reason is that blood zinc levels are less sensitive to zinc deficiency, and it may be difficult to identify a marginal deficiency of zinc. Nevertheless, it still suggests that zinc supplementation may be a way to improve the symptoms of IBS, as it has been found to normalize the copper–zinc ratio and improve symptoms in anxiety and in autism spectrum disorders with concurrent gastrointestinal symptoms [91,93].

There is no obvious intestinal inflammation in IBS patients, but the number and activation of mast cells (MCs) increase as the intestine is stressed [94]. The main structural feature of mast cells is a large number of basophilic granules, which are rich in a variety of bioactive substances; these basophilic particles release various inflammatory mediators, such as histamine and 5-HT, after their activation [95]. One study found that histamine sensitizes the nociceptor transient reporter potential channel V1 (TRPV1) and contributes to IBS symptoms of visceral hypersensitivity in animals [96]. The alterations to 5-HT signaling can result in IBS symptoms such as gastrointestinal dysmotility, visceral hypersensitivity, and abnormal intestinal secretion motility [97,98]. The expression of 5-HT and its receptor in the intestinal mucosa of IBS patients were higher than the normal, and 5-HT receptor antagonist therapy can relieve IBS-D symptoms effectively [99,100]. The microstructure of MCs showed that its granules are rich in zinc [101]. MCs receptor FcεRI stimulation induced an increase in intracellular free zinc [102]. The zinc chelator, N,N,N,N-tetrakis (2-pyridylmethyl) ethylenediamine can significantly inhibit the degranulation of FcεRI and the production of cytokine production such as TNF-α and IL-β [103]. These subjects suggest that zinc figures prominently in the activation of MCs, and it may regulate the degranulation of mast cells to affect the release of bioactive substances from mast cells and mediate the occurrence of IBS symptoms.

As a functional intestinal disease, IBS is closely related to the psychological and mental health factors of patients; hence, the dysfunction of the brain–gut axis is increasingly believed to be one of the main mechanisms underlying IBS. Stress and emotion trigger neuroimmune and neuroendocrine responses through the brain–gut axis, thus affecting the gastrointestinal tract and inducing IBS symptoms. Zinc is related to the secretion of neurotransmitters. Zinc deficiency can critically impair the GABAergic neurotransmitter system [104], while it is generally recognized that GABA plays a regulatory role in gastrointestinal motility and visceral sensation [105]. In addition, zinc homeostasis also affects the transmission of neurotransmitters, including 5-HT, norepinephrine, dopamine, etc. [106,107], and these neurotransmitters contribute to the pathogenesis of IBS by activating or inhibiting the central or intestinal nervous system [108,109]. As stated in Section 3.2, zinc homeostasis affects the structural and functional integrity of the intestinal mucosa, and intestinal mucosal permeability and intestinal microbial composition are also hypothetical etiological mechanisms of IBS [85]. In summary, zinc may regulate the central nervous system and gastrointestinal system by affecting the intestinal mucosal barrier function, mast cell activation, and neurotransmitter secretion, relative to the initiation and development of IBS.

### 4.3. Colorectal Cancer

CRC is a common malignant tumor of the digestive tract with high morbidity and mortality, and it is the third deadliest cancer in the world. The etiology of CRC is relatively clear; dietary factors, genetic factors, and pathological factors are the main causes of CRC, but the mechanism of its occurrence and development is not yet clear.

Whether there is a correlation between CRC and zinc homeostasis is controversial. It has been found that there is no substantial association between serum zinc levels and rectal cancer in the representative general population in the USA [110]. However, in Iran’s CRC population, a survey found that serum zinc levels were significantly lower than in healthy individuals [111]. Christudoss et al. [112] found that in rats with colon cancer induced by dimethylhydrazine, the decrease in plasma zinc, tissue zinc, and the activity of zinc-related enzymes was associated with the development of precancerous lesions, and these biochemical parameters further decreased with the progression to carcinoma in the colon. In addition, there is evidence that zinc intake has an effect on the risk of CRC among a certain demographic. A meta-analysis found a significant correlation between zinc intake and CRC risk in female patients but not in male. In addition, it was found that zinc intake was significantly associated with the risk of colorectal cancer in Asia, but not in the USA and Europe [113]. Further, Li et al. [113] found a U-shaped relationship (the cut-point dose was 22 mg/d) between the dose of zinc intake and colorectal cancer risk. In vitro studies also found that excessive Zn^2+^ induced epithelial transformation through the GSK3/mTOR signaling pathway to promote the migration and invasion capabilities of CRC cells [114]. This indicates that both too much and too little zinc intake can increase the risk of CRC.

Zinc deficiency increases the production of reactive oxygen species (ROS) and the sensitivity to oxidative damage [115,116,117], causing antioxidant dysfunction and resulting in an increased possibility of tumorigenesis. Zinc supplementation can effectively alleviate oxidative stress [115] and prevent oxidative DNA damage in cells to maintain the integrity of DNA in cells [118,119,120]. In addition, zinc participates in DNA transcription and replication through zinc finger protein and the p53 tumor suppressor protein, and so on, and zinc deficiency can affect the active DNA-binding expression of numerous transcription factors that are involved in cancer promotion and progression, such as AP-1, NF-κB, and p53 [118,121], which can lead to increased DNA damage, mutation, genomic instability, and increased inflammatory levels in the body, resulting in an increased risk of cancer.

Although it is still controversial whether the level of zinc can change in patients with CRC, improper intake of zinc does increase the risk of cancer, such as CRC, which indicates that the change in zinc homeostasis is indeed related to the occurrence and development of CRC. Hence, it is not surprising that researchers have focused on the role of zinc transporters. Zinc is involved in various life processes such as the proliferation and migration of tumor cells, and the maintenance of intracellular zinc levels is of great significance to the life progress of tumor cells. Sheng et al. [122] found that the expression of ZIP7 in the colon tissue of CRC patients was significantly increased, and ZIP7 could mediate the extracellular influx of Zn^2+^. It was also shown that knockdown of ZIP7 interfered with cell cycle progression and induced G2/M cell cycle arrest, as well as boosting apoptosis in colorectal cancer cells [122]. Similarly, Barresi et al. [123] found differential gene expression of ZnT and ZIP transporters in CRC cells to maintain high zinc turnover predominantly required in the ER vesicles of tumoral cells where appropriate folding, assembly, and modification of proteins occur.

To summarize, zinc is a necessary trace element for the resistance to CRC; zinc deficiency can lead to DNA damage, impair antioxidant function, and increase the risk of cancer. Zinc transporters are also involved in the development of CRC through the maintenance of intracellular zinc levels in the CRC cells. This also suggests the importance of maintaining zinc homeostasis in the prevention and treatment of CRC.

### 4.4. Celiac Disease

Celiac disease (CeD) is a kind of chronic intestinal malabsorption syndrome caused by gluten-intake intolerance in genetically susceptible individuals [124]. It is an autoimmune disease, typically characterized by chronic diarrhea, abdominal pain, and other gastrointestinal symptoms [125] and often leads to growth retardation in children [126]. At present, the pathogenesis of CeD is not completely clear. It is mainly believed that CeD is the result of the interaction of genetic, immune, and environmental factors [127].

A recent study of a U.S. population found that serum zinc levels were decreased significantly in children aged 6–19 years old with celiac disease, but there was no significant change in patients over 20 years old [128]. In fact, zinc deficiency is common in patients with celiac disease [129]. The possible reason for zinc deficiency in patients with CeD is that the atrophy of the proximal intestinal villiin in CeD patients results in malabsorption of zinc [130]; in addition, the chelation of zinc by fatty acids, excessive loss due to protein-losing enteropathy, or excessive utilization due to enhanced enterocyte turnover can also led to zinc deficiency in CeD patients [129]. The symptoms of slower growth, later sexual maturity, and slower wound healing, as well as intellectual disabilities in patients with CeD may be related to zinc deficiency. Although zinc deficiency is common in patients with CeD, there is no evidence that the imbalance in zinc homeostasis is the cause of CeD. However, because zinc and zinc homeostasis are conducive to the maintenance of intestinal mucosal barrier function, they could be a therapeutic modality in CeD by way of reducing gluten and other luminal antigen permeation across the small bowel epithelium of CeD patients.

## 5. Conclusions

Zinc and zinc homeostasis strongly contribute to the maintenance of the intestinal mucosal barrier structure and function. An imbalance in zinc homeostasis can cause impairment of the epithelial barrier function, altered immune responses, and dysbiosis of the gut microbiota, and it is related to the occurrence and development of intestinal diseases such as IBD, IBS, and CRC. However, whether the improvement of intestinal barrier function with zinc can alleviate and treat these intestinal diseases still needs further clinical verification, and the molecular mechanism by which zinc regulates these intestinal diseases also remains poorly understood; Moreover, the function of zinc transporters has not been clearly discussed. More experimental and clinical studies are needed to further specify the precise role of zinc and zinc homeostasis in intestinal diseases, so as to provide new methods of intestinal disease diagnosis and therapy.
